# Retrospective evaluation of sirolimus (mTOR blocker) for lymphatic malformations presenting in the neonatal period: a single-center experience

**DOI:** 10.3389/fped.2026.1900903

**Published:** 2026-10-07

**Authors:** Alexey Podkamenev, Arina Syrtsova, Gleb Kondratiev, Victor Nabokov, Roman Ti, Svetlana Kuzminykh, Vladislav Dvoretsky, Ani Ajamoghlian, Alexander Kostylev, Irina Myznikova

**Affiliations:** 1 Department of Surgical Diseases of Childhood Named After G.A.Bairov, St. Petersburg State Pediatric Medical University, St. Petersburg, Russia; 2 Department of Oncology, Pediatric Oncology and Radiation Therapy, St. Petersburg State Pediatric Medical University, St. Petersburg, Russia; 3 Perinatal Center of the St. Petersburg State Pediatric Medical University, St. Petersburg, Russia; 4 Department of Oncohematology, St. Petersburg State Pediatric Medical University, St. Petersburg, Russia

**Keywords:** lymphatic malformation, neonate, sirolimus, mTOR, vascular anomalies, conservative therapy

## Abstract

**Background:**

Lymphatic malformations are rare vascular anomalies. Currently, there is no uniform approach to the treatment of these patients. Neonates with extensive disease often suffer from multiple complications, organ dysfunction, and various tissue lesions, which may be life-threatening and lead to aesthetic deformity. Treatment with sirolimus has the potential to improve symptoms and downsize the lesion, up to complete regression. This retrospective single-center study presents our experience with oral sirolimus in the treatment of newborns with lymphatic malformations, evaluating its efficacy and safety.

**Methods:**

A retrospective analysis was conducted on 23 patients in the neonatal period and early infancy with lymphatic malformations, treated at the St. Petersburg State Pediatric Medical University between 2021 and 2025. Seven patients underwent primary radical surgical treatment and were not included in the analysis. The final study cohort consisted of 16 patients who received primary conservative therapy with sirolimus. Fifteen out of 16 patients started receiving oral sirolimus before the age of 28 days of life. The median age at the commencement of treatment was 15 days after birth. After instrumental and laboratory examination, sirolimus was administered at a starting reduced dose of 0.5 mg/m^2^/day divided into two daily doses, followed by a gradual increase under clinical and laboratory monitoring. The therapeutic concentration of sirolimus in the blood (5–15 ng/mL) was monitored once a week.

**Results:**

Disease stabilization or regression was achieved in all 16 patients (100%), with a partial or complete radiological response (≥50% volume reduction) in 13 patients (81.25%) and complete regression (>90% volume reduction) in 10 patients (62.5%) at the 6-month assessment. No treatment-related serious adverse events requiring permanent discontinuation were observed. Recorded adverse events included transient respiratory infections (*n* = 2), diarrhea (*n* = 2), aphthous stomatitis (*n* = 1), and mild anemia (*n* = 1), all of which resolved with supportive care or temporary dose adjustment.

**Conclusion:**

In this selected cohort, sirolimus demonstrated an acceptable safety and efficacy profile, supporting its feasibility as a conservative first-line strategy in neonates with extensive or unresectable lymphatic malformations. However, larger prospective studies are needed to confirm these findings.

## Graphical Abstract

**Figure:**
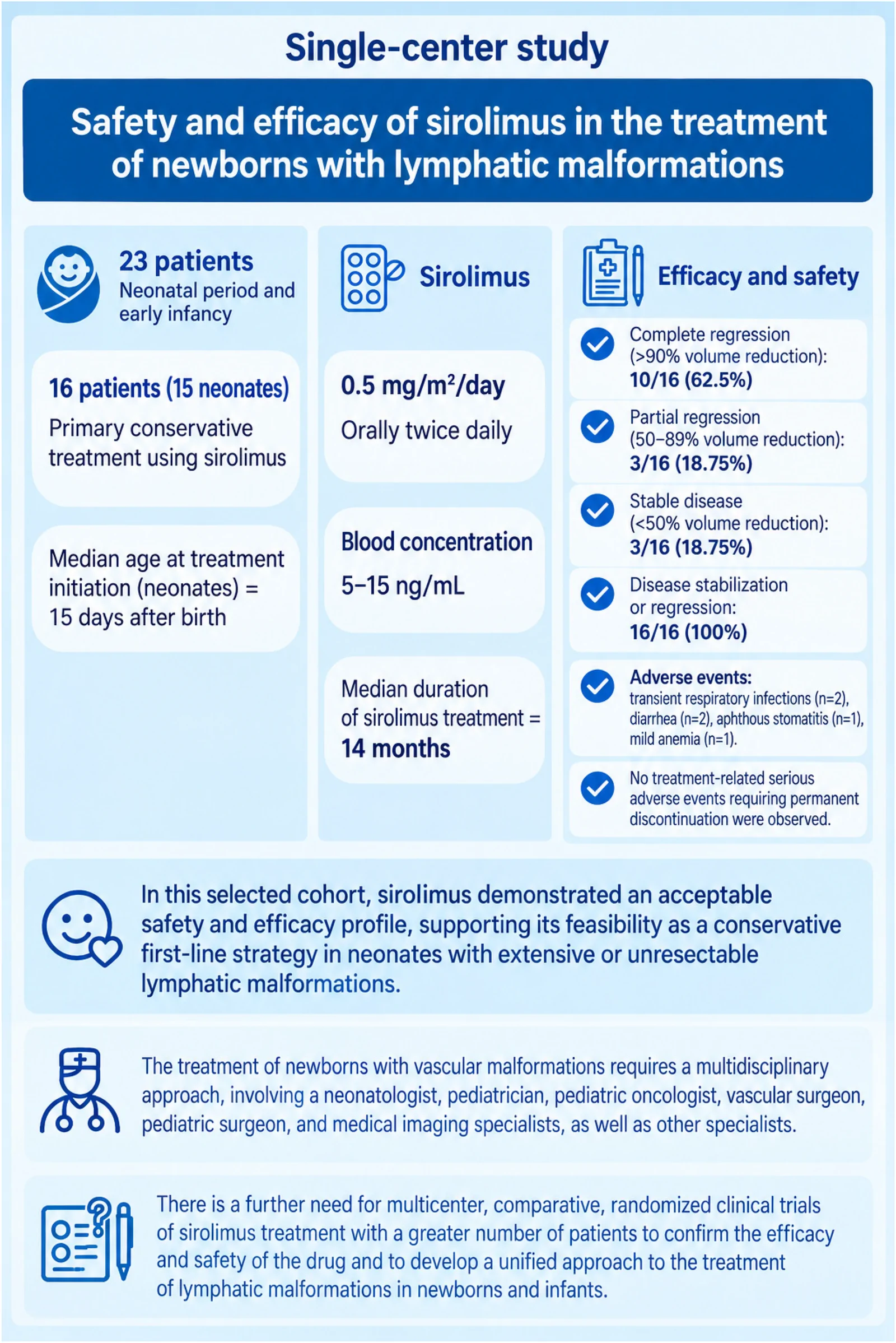

## Introduction

In accordance with the international classification of ISSVA (International Society for the Study of Vascular Anomalies), lymphangiomas are lymphatic vascular malformations (LM) presented as cystic vascular abnormalities without tumor proliferation of endothelial cells (1).

LMs are the second most prevalent type of vascular malformations, following venous malformations (2). The incidence of lymphatic malformations is about 1.2–2.8 per 1,000 live births. Approximately 50%–60% of cases are detected antenatally or immediately after birth. Around 48% of lesions occur in the head and neck, followed by trunk and extremities (42%), abdomen and thorax (10%) (3, 4).

Lymphatic malformations can manifest in a multitude of ways, leading to a diverse range of clinical presentations. Particular mass in the head and neck may cause major cosmetic and functional problems or even compression of the main airway (5–10).

The most likely pathogenetic mechanism of lymphatic vascular abnormalities is mutations in the PIK3CA gene, which leads to abnormal functioning of endothelial cells and disruption of the structure of lymphatic channels. Mutations in this gene have been identified in the majority (about 80%) of common cystic lymphatic malformations (11).

There are no standardized treatment protocols for patients with lymphatic malformations. The frequent proximity of lymphatic malformations in the head and neck to vital structures poses significant challenges in the treatment. Historically, surgical excision has been the standard of care. Radical surgical correction of lymphatic malformations can lead to technical difficulties associated with extensive damage to tissues and organs. It also causes relapses and life-threatening complications. Complete excision of LM is often difficult to achieve and is accompanied by high level of recurrence. According to Huerta CT et al., rate of complications of surgical treatment for LM account for 26.6% (range 0%–100%) and recurrence is 13.2%. Other methods of treatment of lymphatic malformations include sclerotherapy, radiofrequency ablation and laser treatment with varying rates of treatment success. The effectiveness of LM sclerotherapy is up to 84.2% when using doxycycline and up to 40% when treating with bleomycin (7, 12–14).

The first reports of conservative treatment of vascular malformations with rapamycin (sirolimus) in children appeared in 2008–2011 (5, 15, 16).

Rapamycin (sirolimus) is a natural antibiotic produced by the bacterium Streptomyces hygroscopicus, which was first introduced in the literature in 1975 as a fungicidal agent (17). Rapamycin in the cell combines with the protein FKBP-12 (immunophilin) and inhibits the main enzyme of cell cycle regulation protein kinase mTOR (mechanical/mammalian target of rapamycin). Kinase mTOR inhibits the cell cycle between periods of resting and synthesis phase. Activation of the PI3 K/AKT pathway leads to activation of mTORC1. Diseases associated with disorders in the PI3K->AKT->mTOR signaling pathway, including various vascular malformations, are successfully treated with sirolimus (18–21).

Sirolimus is usually prescribed in a starting dose of 1–1.6 mg/m^2^/day orally for 2 times (every 12 h). The therapeutic range of the drug concentration in the blood, minimizing toxic and undesirable effects, is 5–15 ng/mL. Side effects include: transient recurrent neuropathy, hypercholesterolemia, hypertriglyceridemia, aphthous stomatitis, mucositis, mild hypertension, increased liver enzymes, functional dyspepsia, nausea/vomiting, diarrhea, neutropenia, lymphopenia, thrombocytopenia, hypophosphatemia, eczema (10, 12).

Sirolimus accumulates in the blood cells, reaches its maximum concentration after a single dose in 60 min and has a half-life of 62 +/−16 h. The coefficient reflecting the ratio of concentration in blood to concentration in plasma is 36 (36:1, blood: plasma). The drug is mainly excreted through the gastrointestinal tract with bile (91%) and, to a lesser extent, by the kidneys (2.2%).

Rapamycin undergoes intensive metabolism under the influence of the CYP3A4 isoenzyme in the intestinal wall and liver. It is also a substrate for P-glycoprotein of the small intestine, the system of excretion of many drugs. This should be considered when administering medications that are strong inducers of CYP3A4 (rifampicin, rifabutin, phenobarbital) or strong inhibitors of CYP3A4 and P-glycoprotein (HIV and hepatitis C protease inhibitors, antifungal drugs from the azole group) simultaneously with rapamycin preparations (18–21).

Several case series have reported the use of sirolimus in neonates with lymphatic malformations, but data on dosing strategies, safety monitoring, and long-term outcomes in this specific population remain limited (12, 22–26). To address this gap, we present a relatively large single-center case series of 16 patients characterized by neonatal initiation of oral sirolimus and a broad anatomical distribution of lesions, aiming to evaluate the drug's efficacy and safety in our hospital.

## Methods

### Study design and source population

This study is an uncontrolled retrospective case series based on the review of electronic patient records at the St. Petersburg State Pediatric Medical University. Due to the retrospective and observational nature of the study, treatment allocation was non-randomized and strictly determined by clinical indications and multidisciplinary consensus at the time of the patients’ admission. Electronic databases were searched to consecutively identify patients managed for lymphatic malformations (LMs) in the neonatal period or early infancy between January 1, 2021, and December 31, 2025.

### Inclusion and exclusion criteria

Patients were included in the final analysis if they met the following criteria:

1. age at presentation ≤ 3 months of life;
2. clinical and radiological diagnosis of LM verified by ultrasound, CT, or MRI;
3. primary conservative therapy with oral sirolimus initiated at our institution.

During the chart review, patients were excluded from the final analyzed cohort if they: underwent primary radical surgical treatment as their initial therapy (*n* = 7) or had an unverified or altered diagnosis upon histological examination.

A total of 24 electronic records with a presumptive diagnosis of neonatal mass or LM were initially screened. One patient presenting with a neck mass was excluded prior to establishing the source population because a formal biopsy and morphological examination established a definitive diagnosis of teratoma. Thus, the baseline source population consisted of 23 consecutively treated infants with verified LMs (Figure 1).

**Figure 1:**
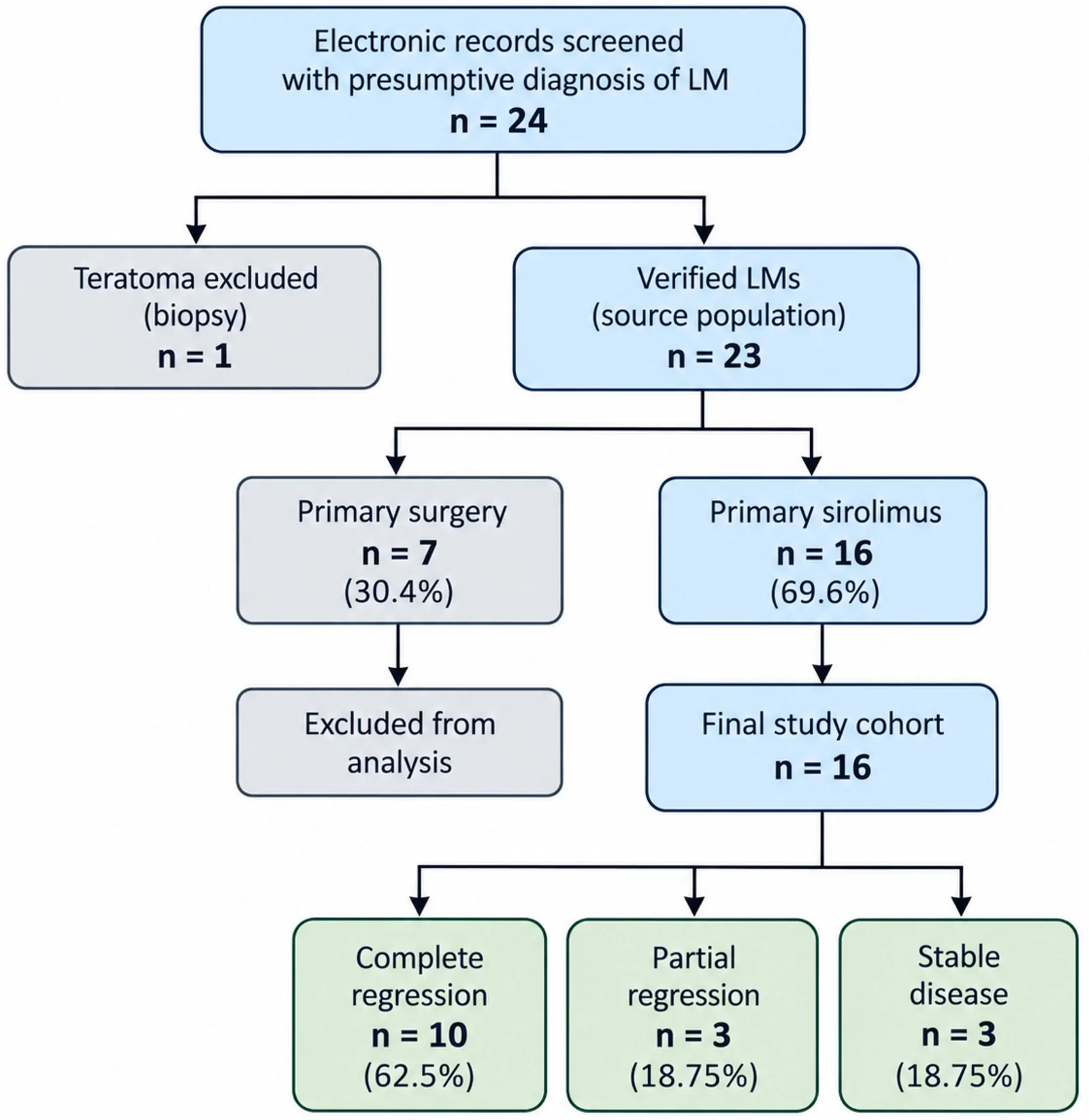
Patient flow diagram.

### Characterization of treatment allocation

Treatment allocation was determined by a multidisciplinary team based on lesion anatomy and clinical severity. Primary radical surgery was chosen for 7 out of 23 patients (30.4%) who presented with localized, well-demarcated lesions where complete excision carried minimal risk to vital structures. Conversely, primary conservative therapy with oral sirolimus was initiated in 16 patients (69.6%) presenting with extensive, infiltrative, or complex LMs where surgical intervention carried high morbidity or mortality risk. Other modalities, such as primary sclerotherapy or active observation, were not utilized during this study period.

Within the final sirolimus cohort (*n* = 16), 15 patients initiated therapy during the neonatal period (median age: 15 days). All 16 patients had complete medical records with clinical and laboratory data, and no patients were lost to follow-up or discontinued treatment due to toxicity.

### Diagnostic workup and treatment protocol

The complex diagnostic approach included a clinical examination and medical imaging (ultrasound, CT, MRI). In patients with abdominal lymphangioma, diagnostic laparoscopy was performed to confirm the diagnosis. All patients were consulted by a pediatric oncologist, a pediatric surgeon, and an angiosurgeon.

Sirolimus was initiated in infants with lymphatic malformation upon diagnosis confirmation, at a starting dose of 0.5 mg/m^2^/day orally in two divided doses, with daily clinical and weekly laboratory monitoring. The target concentration of sirolimus in the blood was 5–15 ng/mL. During hospitalization, monitoring was performed weekly.

Since the start and throughout the entire period of therapy, patients were monitored for basic clinical and laboratory parameters on a weekly basis to assess the safety of the drug in this age group. Medical imaging was performed no earlier than 1–2 months after the start of therapy, or in the case of marked local changes in the area of lymphangioma.

The patients were examined once a month after transition to the outpatient stage of treatment. Long-term outpatient follow-up was performed by a pediatric oncologist, pediatrician, pediatric surgeon, or angiosurgeon.

Table 1 summarizes the clinical and anamnestic data of all patients with lymphatic malformations treated during the study period.

**Table 1:** Clinical and anamnestic data of patients with lymphatic malformations treated for the period 2021-2025.

№	Antenatal diagnosis	Gestation week	C-section/vaginal birth	Sex	Weight, g	Height, cm	Apgar point	Localization	Beginning of sirolimus	Response	Age at which the operation was performed	Side effects
1	no	37	C-section	f	-	-	6/7	Neck	-	-	Radical 1 m	-
2	3 trimester	39	C-section	m	4,240	55	8/9	Neck	-	-	Radical 11 day of life	-
3	2 trimester	39	C-section	m	3,680	52	8/9	Mesentery of the small intestine	-	-	Radical 1 m	-
4	3 trimester	39	C-section	m	3,300	51	8/9	Upper limb	-	-	Radical 13 day of life	-
5	3 trimester	39	C-section	f	4,320	54	8/9	Neck	-	-	Radical 1.5m	-
6	3 trimester	38	Vaginal birth	f	3,070	50	8/9	Mesentery of the small intestine	-	-	Radical 22 day of life	-
7	3 trimester	37	C-section	f	2,830	49	8/9	Neck	-	-	Radical 22 day of life	-
8	3 trimester	39	C-section	m	3,450	51	9/9	Head,neck	21 day of life	Mild regression, stabilization	No	Anemia, respiratory infections
9	2 trimester	39	C-section	f	4,050	54	8/9	Head,neck	11 day of life	Mild regression, stabilization	Secondary operation 1.5 y	Respiratory infections, diarrhea
10	no	39	C-section	m	3,300	49	8/9	Neck	25 day of life	Complete regression	No	No
11	3 trimester	41	C-section	m	4,090	54	8/9	Head,neck	16 day of life	Complete regression	No	No
12	3 trimester	36	C-section	f	2,020	47	7/7	Mesentery of the small intestine	15 day of life	Complete regression	Diagnostic laparoscopy	No
13	No	39	C-section	f	4,110	53	8/9	Thorax	15 day of life	Complete regression	No	Aphthous stomatitis
14	3 trimester	32	C-section	m	2,880	44	7/7	Thorax,limbs	1.5 m	Complete regression	Biopsy	No
15	2 trimester	40	Vaginal birth	f	3,420	53	8/9	Mesentery of the small intestine	11 day of life.	Complete regression	Diagnostic laparoscopy	No
16	2 trimester	40	Vaginal birth	f	3,760	53	8/9	Mesentery of the colon	21 day of life	Complete regression	Diagnostic laparoscopy	No
17	3 trimester	37	C-section	m	3,280	52	7/8	Neck,thorax	15 day of life	Mild regression, stabilization	No	No
18	No	35	Vaginal birth	m	2,430	47	7/8	Abdominal cavity, hyloperitoneum	2 day of life	Complete regression	No	No
19	3 trimester	38	C-section with exit-procedure	m	3,450	50	6/7/8	Head, neck	1 day of life	Complete regression	No	Diarrhea
20	3 trimester	39	Vaginal birth	f	3,100	50	9/10	Retroperitoneal, lower back	8 day of life	Complete regression	No	No
21	2 trimester	38	C-section	m	3,980	50	8/9	Neck	14 day of life	regression	No	No
22	3 trimester	37	C-section	m	2,930	49	8/9	Abdominal cavity	23 day of life	regression	Diagnostic laparoscopy	No
23	3 trimester	37	C-section	m	3,970	54	8/9	Chest and abdominal Subcutaneous adipose tissue	10 day of life	regression	No	No

Antenatal diagnosis was recorded when available from the medical charts, but detailed prenatal imaging data were not consistently documented for all patients due to the retrospective nature of the study.

### Response assessment

Given the retrospective nature of this study, response assessment was based strictly on the review of clinical documentation and follow-up imaging studies (ultrasound, CT, or MRI) available in the electronic medical records, closest to the 6-month time point after treatment initiation. Lesion volumes were estimated using the standard ellipsoid formula (length×width×thickness×0.52) based on multi-planar radiologist measurements.

To ensure reproducibility, the original clinical descriptions used in Table 1 were retrospectively harmonized with objective volumetric changes as follows:

1. “complete regression” was defined as a near-complete or complete disappearance of the lesion, corresponding to a ≥ 90% reduction in estimated volume;
2. “regression” (partial response) corresponded to a 50% to 89% volume reduction; and
3. “mild regression/stabilization” (stable disease) was defined as a less than 50% volume reduction without objective disease progression.

In strict accordance with the retrospective design, no independent or blinded prospective radiological review was performed, and response categories reflect the validated entries within the institutional database.

## Results

In our clinic, 16 patients with lymphatic malformations received primary treatment with sirolimus. At the beginning of treatment, the target concentration of sirolimus in the blood (5–15 ng/mL) was achieved using a minimum dose of 0.5 mg/m^2^/ day orally in 2 doses. The dose was further increased to 1.0 mg/m^2^/day as the target concentration decreased. This correlated with the child's weight gain.

Table 2 shows the laboratory data, showing the minimum and maximum levels of each indicator for the entire follow-up period: a biochemical blood test indicating liver and kidney markers of toxicity, a clinical blood test, and data from a basic instrumental cardiological examination and examinations by specialists.

**Table 2:** Clinical and laboratory parameters of patients receiving sirolimus.

Data	1	2	3	4	5	6	7	8	9	10	11	12	13	14	15	16
ALT	17.0–43.0	12.0–14.0	23.3–37.7	23.0–25.8	17.3–23.4	11.0–16.0	24.7–30.0	14.7–30.6	11.7–19.9	14.9	40.1–84.0	19.8–29.4	6.5–73.0	18.0–19.3	13.4–21.5	36.8–28.9
AST	20.0–57.0	19.0–45.0	37.7–42.1	50.0–57.0	25.2–37.3	25.0–51.0	24.4–44.2	35.5–61.5	25.6–49.6	28.1	37.5–116.6	26.8–33.8	17.5–91.9	32.5–34.5	21.7–66.1	29.8–36.6
Total bilirubin mmol/L (direct/indirect)	8.8–20.0 (6.9–8.3/ 2.0–11.7)	7.0–109.0 (2.9–9.3/ 4.3–104.3)	5.0–7.0 (2.3–3.5/2.7–3.5)	19.0–165.0 (6.8–7.6/ 13.06–157.4)	3.12–32.6 (3.45–5.9/ 2.6–26.7)	7.0–126.0 (2.8–11.2/ 3.3–120.0)	24.0–205.0 (3.4–10.4/ 18.3–194.6	38.3–63.7 (4.4–7.9/ 33.9–55.1)	2.1–23.9 (0.55–7.7/ 1.2–16.2)	205.0 (13.3/ 191,7)	10.7–44.6 (8.1–9.16/ 8.1–36.4)	27.2–59.8 (2.56–10.5/ 26.4–49.3)	7.9–200.0 (6.2–20.2/ 4.9–187.6	127.0–139 (7.0–10.3/ 120–128.7)	79.0–200.3 (9.0–15.0/ 70.0–185.3)	177,6–213–5 (12,3–14,0/ 165.3–199,5)
Creatinine mmol/l	0.025–0.049	0.031–0.052	0.031–0.039	0.062–0.037	0.026–0.045	0.035–0.038	0.017–0.032	0.025–0.039	0.024–0.050	0.065	0.040–0.045	0.029–0.035	0.031–0.076	0,043–0,064	0,035–0,053	0,038–0,056
Urea mmol/l	1.0–1.7	2.0–3.5	2.8–4.1	3.0–3.3	1.8- 4.7	2.0–4.5	3.9–5.9	2.6–3.9	4.3–5.6	5.6	1.5–2.4	3.6–4.59	4.6–7.59	3,3–4,0	0,6–0,6	4,1–5,1
Triglycerides mmol/l	0.85–9.12	-	-	-	-	0.7	-	-	-	-	-	-	0.2–1.38	-	-	-
Phosphorus mmol/l	2.1–2.2	2.08–2.35	-	-	2.03–2.13	2.25–2.46	-	-	1.9–2.1	-	-	-	1.87–2.01	-	-	-
Lymphocyte level is 10^9/l	1.4–7.9	3.6–8.4	5.1–6.4	4.3–5.3	3.9–6.55	3.3–6.0	5.1–6.9	3.8–4.6	3.2–6.2	6.2	7.2–8.8	8.8–9.7	2.8–4.7	1,4–3,8	4,0–4,66	4,73
Neutrophil level 10^9/l	1.9–6.6	8.1–26.6	1.9–2.4	1.8–4.2	1.8–4.08	1.3–6.9	0.59–13.7	1.5–4.2	2.1–6.1	5.1	1.5–8.3	4.1–6.9	2.9–26.6	1,4–6,0	1,0–1,09	2,74
Platelet count 10^9/l	218–509	284–562	308–350	363–472	485–727	338–576	232–530	182–382	175–461	512	395–690	472–538	349–696	369–371	277–404	358–555
Blood pressure	Normal															
ECG, ECHO	No specific changes															

Laboratory parameters showed occasional deviations, which were transient and resolved without specific intervention. No treatment-related serious adverse events requiring permanent discontinuation were observed. Recorded adverse events included transient respiratory infections (*n* = 2), diarrhea (*n* = 2), aphthous stomatitis (*n* = 1), and mild anemia (*n* = 1), all of which resolved with supportive care or temporary dose adjustment.

Histological examination was performed only in cases where the diagnosis was uncertain. In the study cohort, biopsy was performed in one patient initially suspected of having a cervical lymphangioma, which revealed a teratoma; this patient was excluded from the analysis. In all other cases, the diagnosis was confirmed by imaging studies. Genetic testing for PIK3CA mutations was not routinely performed at our institution during the study period, consistent with the retrospective nature of this analysis.

## Discussion

The initial utilization of sirolimus for vascular malformations was documented in early case reports demonstrating significant clinical improvement in pediatric patients (27). Subsequently, accumulating evidence has established the efficacy of this agent in reducing lesion volume and mitigating symptoms in children with complex vascular anomalies (16, 20). More recently, clinical interest has shifted toward evaluating targeted therapy specifically within the neonatal period. Several case series have reported successful first-line sirolimus use in neonates with extensive head and neck lesions requiring respiratory support, with significant clinical improvement in all cases (22). Pharmacokinetic challenges in this population have also been characterized, showing that standard pediatric dosing protocols often result in supratherapeutic drug levels, thereby necessitating modified dosing regimens (23). Clinical observations in infants under one year of age have confirmed that a starting dose of 0.5 mg/m^2^/day is sufficient to maintain therapeutic trough concentrations (24). More recently, substantial volumetric reductions have been documented in neonates with life-threatening or extensive cervicofacial lesions, with one series reporting a mean volume reduction of 79% in patients treated with sirolimus combined with sclerotherapy (25, 26).

Our study extends these observations to a notably broad anatomical spectrum, including not only head and neck lesions, but also thoracic, abdominal, mesenteric, and extremity involvement—sites where surgical resection often carries significant morbidity and technical challenges. With a median treatment initiation age of 15 days and a starting dose of 0.5 mg/m^2^/day, titrated to a target trough concentration of 5–15 ng/mL, we observed stable disease stabilization or regression in 100% of our patients. Specifically, complete regression was achieved in 10 out of 16 cases (62.5%), partial regression in 3 cases (18.75%), and disease stabilization in the remaining 3 cases (18.75%) at the 6-month assessment. Importantly, no treatment-related serious adverse events requiring permanent discontinuation were observed. The recorded side effects were mild and manageable, including transient respiratory infections (*n* = 2), diarrhea (*n* = 2), aphthous stomatitis (*n* = 1), and mild anemia (*n* = 1), all of which resolved with supportive care or temporary dose adjustment.

The role of sirolimus in the management of lymphatic malformations must be considered within the broader context of available treatment options. Surgical excision has historically been the standard of care, but it is associated with significant challenges, particularly in anatomically complex locations. According to a recent systematic review by the APSA Outcomes and Evidence-Based Practice Committee, the complication rate of surgical treatment for LMs is 26.6% (range 0%–100%), with a recurrence rate of 13.2% (12). Sclerotherapy offers an alternative with variable success rates, ranging from 40% with bleomycin to 84.2% with doxycycline (12). In this context, sirolimus provides a valuable conservative option, particularly for neonates with extensive or unresectable lesions where surgical intervention carries high morbidity or mortality risk.

In our cohort, five patients presented with abdominal or mesenteric LMs — sites where surgical resection poses substantial technical challenges and risks of intestinal injury, malabsorption, or short bowel syndrome. Diagnostic laparoscopy was performed to confirm the diagnosis in these patients, but definitive surgical resection was deferred in favor of sirolimus therapy. This approach aligns with the concept of neoadjuvant conservative therapy, which can reduce surgical risks by stabilizing lesion size or achieving sufficient regression to allow for a less extensive procedure (28). In one patient, sirolimus therapy resulted in only mild regression with subsequent stabilization, and secondary surgical intervention was performed at 1.5 years of age. This case illustrates that sirolimus does not always eliminate the need for surgery, but may facilitate a safer, more elective surgical procedure at a more favorable age.

The multidisciplinary management of neonates with vascular malformations is essential, requiring collaboration among neonatologists, pediatricians, pediatric oncologists, vascular surgeons, pediatric surgeons, and radiologists (7, 10). In our institution, a comprehensive imaging protocol was employed, with ultrasound, CT, and MRI used in combination to confirm the diagnosis, define the anatomical extent of the lesion, and assess its relationship to adjacent structures. This multimodal approach was guided by the technical capabilities of our center and the individualized needs of each patient.

### Limitations

Several limitations of this study should be acknowledged. First, the retrospective, uncontrolled single-center design introduces potential selection bias, as treatment allocation was influenced by lesion location, resectability, and clinical severity. Second, the small sample size (*n* = 16) limits the generalizability of our findings and precludes subgroup analysis to identify predictors of response. Third, the absence of a comparator group (e.g., surgery or sclerotherapy) prevents direct comparison of treatment modalities. Fourth, the relatively short follow-up period (median 18 months, range 6–24 months) does not allow for assessment of long-term durability of response or potential recurrence after sirolimus discontinuation. Fifth, while we applied standardized radiological criteria for response assessment, the imaging intervals and modalities were not uniform across all patients due to the retrospective nature of the study. Finally, adverse events may have been underreported due to the retrospective design and reliance on medical record documentation.

Despite these limitations, our study provides valuable data on the feasibility and safety of sirolimus therapy in a relatively large neonatal cohort with diverse anatomical presentations. The detailed clinical and safety data reported here may inform clinical practice and guide future prospective studies.

## Conclusion

In this selected single-center cohort of neonates with extensive or unresectable lymphatic malformations, clinical and radiological improvement was observed during sirolimus treatment, with disease stabilization or regression in all patients and a partial or complete radiological response in 81.25% of patients at 6 months. The safety profile was acceptable, with no treatment-related serious adverse events requiring permanent discontinuation. These findings support the feasibility of sirolimus as a conservative first-line strategy, potentially deferring or, in some cases, avoiding high-risk surgical intervention. However, the small sample size, uncontrolled design, heterogeneous outcome assessment, and limitations of retrospective safety reporting preclude definitive conclusions regarding efficacy or safety. Multicenter prospective trials with standardized response criteria and long-term follow-up are needed to establish the optimal role of sirolimus in the management of neonatal lymphatic malformations.

## Data Availability

The original contributions presented in the study are included in the article, further inquiries can be directed to the corresponding author.
